# Supplementary material for: Revealing Allosteric Mechanism of Amino Acid Binding Proteins from Open to Closed State
Source: Molecules. 2023 Oct 17;28(20):7139. doi: 10.3390/molecules28207139 (PMC10609312; doi:10.3390/molecules28207139)
Supplement: Supplementary file 1 [file molecules-28-07139-s001.zip › molecules-2590979-supplementary.pdf]

Supplementary material

Revealing Allosteric Mechanism of Amino Acid Binding Proteins from Open to Closed State

Quanshan Shi [1,2,3,†], Ling Liu [1,2,†], Huaichuan Duan [3,†], Yu Jiang [2], Wenqin Luo [2], Guangzhou Sun [2,3], Yutong Ge [2], Li Liang [2], Wei Liu [2], Hubing Shi [3] and Jianping Hu [2,*]

[1] Department of Thoracic Oncology, Affiliated Cancer Hospital, Guizhou Medical University, Guiyang 550025, China; sqs_715@163.com (Q.S.); yzh2602023@163.com (L.L.)
[2] Key Laboratory of Medicinal and Edible Plants Resources Development of Sichuan Education Department, School of Pharmacy, Chengdu University, Chengdu 610106, China; jy94940514@163.com (Y.J.); m13568453654@163.com (W.L.); s19196482291@163.com (G.S.); 13111153368@163.com (Y.G.); liangli_cdu@163.com (L.L.); liuwei@cdu.edu.cn (W.L.)
[3] Laboratory of Integrative Medicine, Clinical Research Center for Breast, State Key Laboratory of Biotherapy, West China Hospital, Sichuan University and Collaborative Innovation Center, Chengdu 610041, China; sumdhc@163.com (H.D.); shihb@scu.edu.cn (H.S.)
* Correspondence: hjpcdu@163.com
† These authors contributed equally to this work.

Table S1. The shortest pathway of crucial residues to I-Loop in the AABPs-open and AABPs-closed systems.

Systems	Possible shortest pathways
GlnBP-open	(1) D10→Y123→V154→V176→G171→A96; (2) A67→L180→G177→G171→A96; (3) G68→Y123→V154→V176→G171→A96; (4) **K115→Y143→N170→G171→A96**; (5) G119→L155→G177→G171→A96; (6)D157→V154→V176→G171→A96
HisJ-open	(1) D11→K201→Q79→Q141→D149→V114→I102; (2) Y14→Q79→Q141→D149→V114→I102; (3) L52→Q79→Q141→D149→V114→I102; (4) S69→E202→D53→Q79→Q141→D149→V114→I102; (5) **L117→Q79→Q141→D149→V114→I102**
LAOBP-open	(1) D11→D32→K44→C45→K62→D91→A96→E123→K98; (2) F52→A96→E123→K98; (3) S70→S184→K188→D75→N129→E123→K98 (4) S72→S184→K188→D75→N129→E123→K98; (5) R77→K76→N129→E123→K98; (6) **L117→R93→E123→A96→K98**
GlnB-closed	(1) D10→K125→T130→K110→V104→V176→A96; (2) A67→A126→T130→K110→V104→V176→A96 (3) G68→A126→T130→K110→V104→V176→A96; (4) **K115→T130→K110→G171→V176→A96** (5) G119→K129→K110→G171→V176→A96; (6) D157→T130→K110→G171→V176→A96
HisJ-closed	(1) D11→C38→Q44→T43→P57→Q79→K189→K186→P104→E107→G134→F159→I102 (2) Y14→G168→E162→P183→T105→E107→G134→F159→I102; (3) L52→R77→K189→K186→P104→E107→G134→F159→I102 (4) S69→C38→Q44→T43→P57→Q79→K189→K186→P104→E107→G134→F159→I102; (5) **L117→R93→P104→E107→G134→F159→I102**
LAOBP-closed	(1) D11→G34→G9→I27→K229→K225→D232→K176→K98; (2) F52→I8→I27→K229→K225→D232→K176→K98; (3) **L117→Q78→K189→E177→K98**; (4) S70→K60→D21→Y223→Q219→D232→K176→K98; (5) S72→A66→I27→K229→K225→D232→K176→K98; (6) R77→D75→K189→E177→K98
